# The Fear of Death

**Published:** 1890-04

**Authors:** 


### THE FEAR OF DEATH.

The first element in the fear of death is an idea of physical pain. It is natural that this should be connected with the idea of death, for in many cases intense pain precedes death. But the two are far from being invariable accompaniments. Intense pain may be followed by life as well as by death. We must distinguish between the fear of pain and the fear of death. Death may be painless. Pain and death do not stand in the relation of cause and effect. One is sometimes the preceding condition of the other, but not a cause. Besides this, the fact must be recognized that death is but a point of time, an instant, a second, and that neither the preliminary process nor the immediate dissolution is constantly attended by pain. Even the worst death may be welcomed as bringing a release from suffering. So let us thrust aside the notion of pain, and keep carefully separated from it the fear of death.

Second is the idea of the mystery of the change. Let us keep closely in mind what death is—it is an instantaneous change. One moment was life, the next was not life. One instant was the exercise of vital energies, the next their total stoppage. One second one was with this world, the next he is gone from it forever. This mystery, unlike pain, is inseparable from death and the idea of death. One cannot think of death and not think of the mystery of the change and the lonesomeness of it. Every one has to encounter it for and by himself.

Third is the idea of that which is beyond death. This idea also is inseparable from the contemplation of the change. Whether one believes in a life beyond the grave, or in annihilation, makes no difference. There is something beyond, and the dread of that mystery

"Puzzles the will,
And makes us rather bear the ills we have
Than fly to others that we know not of."

All these three ideas are connected with death. And yet the change is one that is being encountered every day. There are few who have not seen one die. It is a matter of general knowledge that the number of death beds where the one who was experiencing the change has been unnerved is very small. The dying one is not moved by his loneliness. He does not weep at the separation. What grief he does manifest is more for those who are left than for himself who is going. Whether a weakened vitality blunts his sensibilities, or whether he is prepared for the last great change by unusual strength matters not. There is the fact. When the dying man comes to die at the real and very decisive moment he has no fear of death.

